# Reliability and validity of ultrasound to measure of muscle mass following allogeneic hematopoietic stem cell transplantation

**DOI:** 10.1038/s41598-022-05577-1

**Published:** 2022-01-27

**Authors:** Nao Hashida, Yuma Tada, Masayuki Suzuki, Kumiko Ito, Yuji Kato, Hironari Tamiya, Jun Ishikawa

**Affiliations:** 1 Department of Rehabilitation, Osaka International Cancer Institute, 3-1-69, Otemae, Chuo-ku, Osaka, 541-8567 Japan; 2 Department of Hematology, Osaka International Cancer Institute, Osaka, Japan

**Keywords:** Cancer stem cells, Haematological cancer, Imaging techniques, Quality of life

## Abstract

Allogeneic hematopoietic stem cell transplantation (allo-HSCT) recipients frequently show physical dysfunction due to loss of muscle mass. This study aimed to clarify the reliability and validity of ultrasound in evaluating muscle mass and to analyze the patterns of change in muscle mass before and after allo-HSCT. We conducted a prospective observational study using data from 68 patients who had undergone their first allo-HSCT. We evaluated the thickness of the quadriceps, biceps, and suprahyoid muscle. Three individual evaluators underwent this examination for each muscle before transplantation and on days 30, 90, and 180 after allo-HSCT. Inter-rater reliability was calculated using the interclass correlation (ICC), and the level of correlation between muscle mass measured by ultrasound and psoas muscle mass assessed using computed tomography (CT) was assessed using Pearson correlation. ICC values ranged from 0.897 to 0.977 in the measurement. The correlation scores were 0.730, 0.546 and 0.579 between psoas muscle and the biceps, quadriceps, and suprahyoid muscle. The thickness of the biceps and quadriceps muscle were both significantly decreased after allo-HSCT from baseline. These results showed that the ultrasound technique was a reliable tool for evaluating muscle mass and detecting changes in muscle mass following allo-HSCT.

## Introduction

Allogeneic hematopoietic stem cell transplantation (allo-HSCT) is a curative therapeutic treatment strategy for hematological malignancies. Conditioning regimens, including high-dose chemotherapy and total body irradiation, are necessary not only for the elimination of malignant hematopoietic cells but also for the engraftment of donor-derived hematopoietic stem cells. These highly cytotoxic conditioning regimens frequently result in severe stomatitis, gastrointestinal mucositis, and anorexia in allo-HSCT recipients^1^.

One of the major complications following allo-HSCT is graft-versus-host disease (GVHD), in which recipients mainly receive high-dose corticosteroid therapy, and consequently, they often get trapped in a state of hyper-catabolism. In allo-HSCT recipients, conditions such as stomatitis, mucositis, anorexia, and hyper-catabolism encompass malnutrition and physical dysfunction; that is, sarcopenia, mainly due to loss of muscle mass^2–6^. A previous report showed that 50.6% of patients receiving allo-HSCT had sarcopenia^7^. In addition, two studies showed that sarcopenia is a predictor of overall survival and non-relapse mortality^8,9^. Therefore, it is important to prevent sarcopenia from developing in patients as well as encourage them to maintain their physical function in order to prevent various complications and improve their QOL^6^. However, examining physical function and muscle mass at an appropriate time is difficult because patients are occasionally isolated in a sterile room or are often in poor medical conditions after allo-HSCT^10^.

To date, the usefulness of ultrasound evaluation in evaluating muscle mass and age-related skeletal muscle atrophy in the upper and lower extremities or suprahyoid muscle has been reported in some studies^11,12^. There are a variety of methods for measuring muscle mass. Magnetic resonance imaging (MRI) and computed tomography (CT) are the gold standards for measuring skeletal muscle mass. Measurements of cross-sectional area or whole-body skeletal muscle mass can be precisely done with MRI or CT; however, they are expensive and not portable^13^. Bioelectrical impedance analysis (BIA) and dual energy X-ray absorptiometry (DXA) have been widely and commonly used in the diagnosis of sarcopenia because of their ease of evaluation. However, muscle mass evaluated using BIA can be affected by the device as well as the patient’s skin condition and edema^13^. Because DXA does not directly estimate muscle mass, its measurements are estimated values^14^. On the other hand, an ultrasound machine is portable and can directly measure muscle mass even if the allo-HSCT recipient is isolated in a sterile room or in poor condition. In this study, we examined the masses of three muscles: the quadriceps muscle, biceps muscle, and suprahyoid muscle. Evaluating quadriceps muscle thickness is particularly useful for the assessment of physical activity because of its important role in locomotor activity^15^. The evaluation of the biceps muscle using ultrasound has also been reported as a significant indicator of sarcopenia and is directly associated with instrumental activities of daily living^16^. Regarding the suprahyoid muscle, the styloglossus muscle has been reported not to be affected by sarcopenia^17^. On the other hand, the geniohyoid muscle is not affected by respiratory input stimulation and has been reportedly associated with muscle mass loss with aging^18^. In the previous study that evaluated the muscle mass of the masseter, geniohyoid, biceps and brachialis, and quadriceps femoris muscles to predict sarcopenia, the geniohyoid muscle mass was the best predictor^19^.

The reliability and validity of ultrasound in assessing muscle mass in allo-HSCT recipients have not been fully understood. The aim of this study was to clarify the inter-rater reliability and validity of ultrasound for evaluating muscle mass and to analyze the patterns of change in muscle mass using ultrasound before and after allo-HSCT.

## Methods

### Study design and setting

We conducted a prospective observational study using data from recipients who had undergone their first allo-HSCT between April 2017 and March 2019 at our institute. Ninety-eight recipients underwent allo-HSCT, and 96 of them were enrolled with written informed consent. All recipients received physical therapy from physical therapists and nutrition care by a nutrition support team while they received myeloablative conditioning or reduced-intensity conditioning at the hospital. Physical therapy consisted of resistance training, aerobic exercise, and stretching. These programs started at least a week before the conditioning regimen for an inpatient setting (low-moderate intensity, 20–30 min sessions on weekdays). Recipients with poor physical function at the time of discharge were carefully monitored to assess their physical function. This study has been performed in accordance with the ethical standards laid down in the 1964 Declaration of Helsinki and its later amendments and approved by the ethics committee of the Osaka International Cancer Institute (approval number 1706089013-2).

### Ultrasound examinations

**Figure 1:**
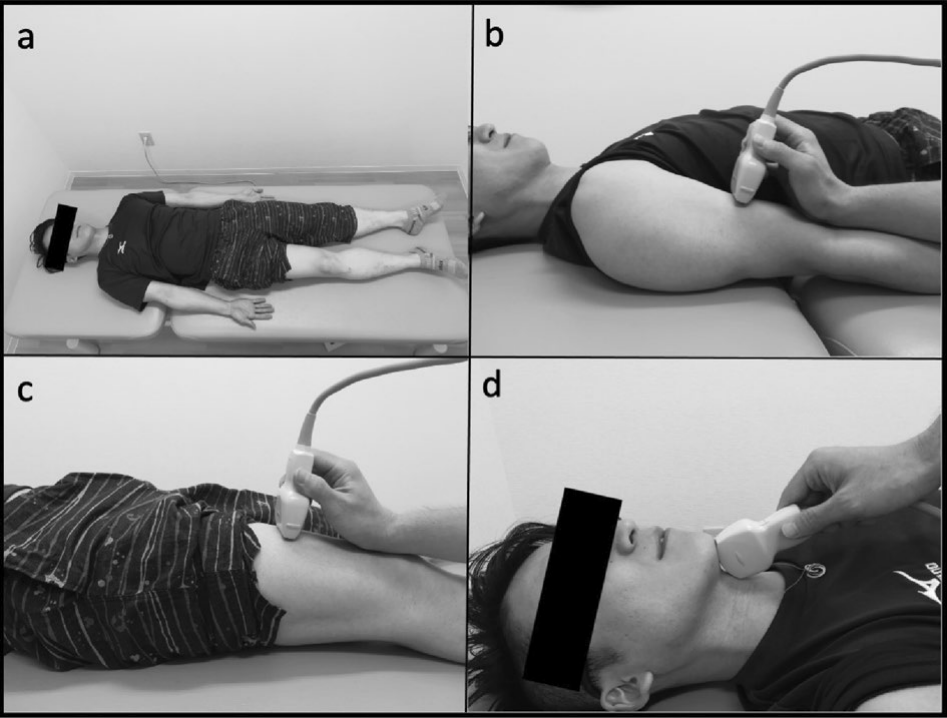
Ultrasound measurement methods. Posture in ultrasound examination (**a**). The landmarks for measurement were as follows: on the 60% distal portion from the acromion to the olecranon of the right arm for the biceps muscle (**b**), on the 60% distal portion from the trochanter major to the epicondylus lateralis of the right thigh for the vastus intermedius muscle and rectus femoris muscle (**c**), and on the middle of the floor of the mouth for the geniohyoid-mylohyoid muscle complex (**d**). All images belong to N.H.

**Figure 2:**
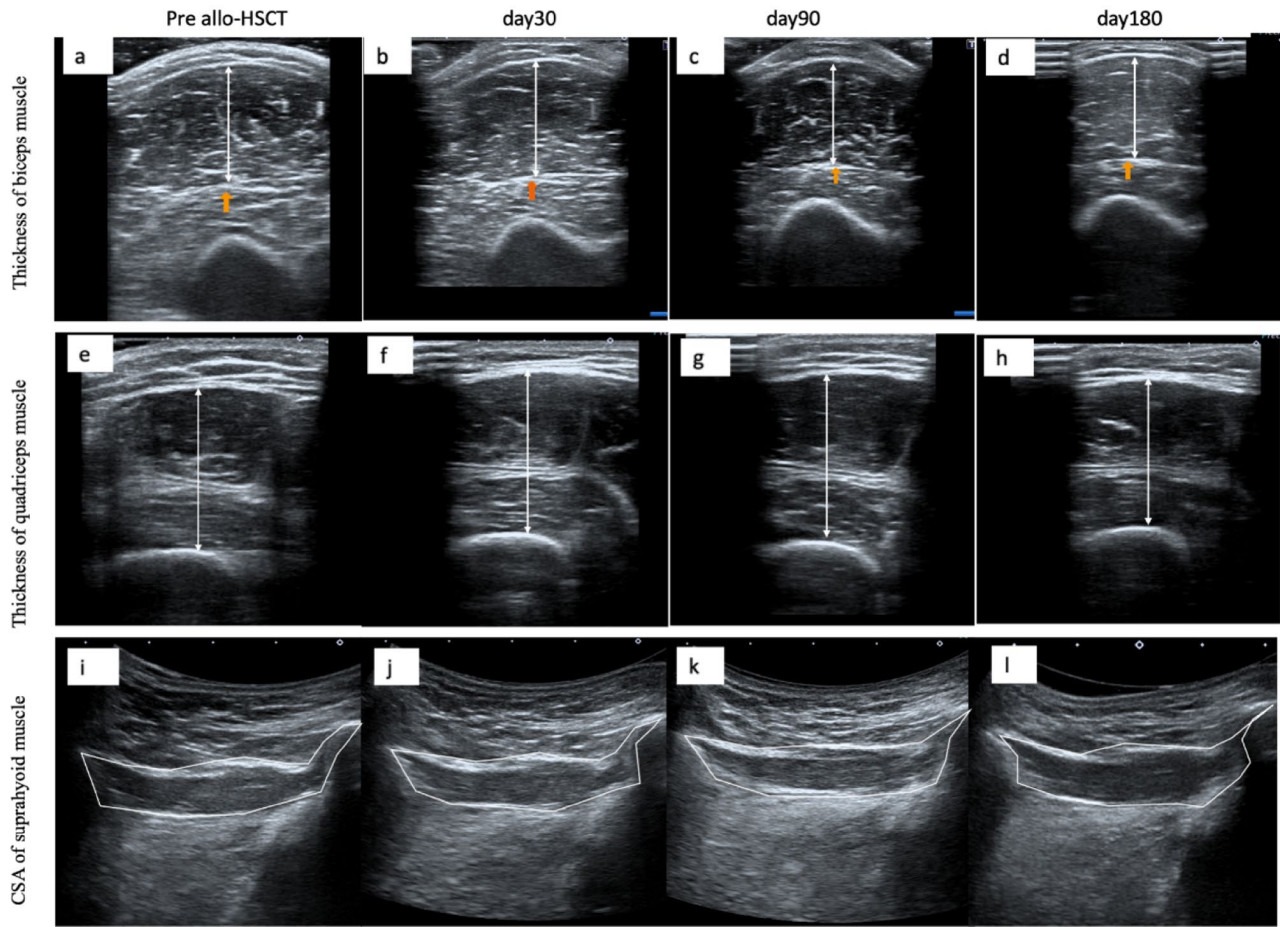
Ultrasound images of the biceps muscle (**a**–**d**), the vastus intermedius muscle and rectus femoris muscle complex (**e**–**h**), and the geniohyoid-mylohyoid muscles complex (**i**–**l**) before and on days 30, 90 and 180 after allo-HSCT, respectively.

Using Aplio 300 (Canon Medical Systems Corporation, Tochigi, Japan), B-mode ultrasound imaging, three examiners evaluated the thickness of the quadriceps and biceps muscles, as well as the cross-sectional area of the suprahyoid muscles. A physician, a physical therapist, and a speech language therapist trained in ultrasound imaging performed the ultrasound imaging within a week before the transplantation and on days 30, 90, and 180 after allo-HSCT. We used a 7.5 MHz linear array probe to assess the thickness of the quadriceps and biceps muscles while a 6 MHz convex array probe was used to assess the cross-sectional area of the suprahyoid muscle. We measured the muscle mass on the right leg, arm, and middle of the floor of the mouth with the patient lying in a supine position and closing the mouth. (Fig. 1a; all images belong to N.H.) The landmarks for measurement were as follows: a 60% distal portion from the trochanter major to the epicondylus lateralis of the right thigh for the quadriceps muscles (Fig. 1b), a 60% distal portion from the acromion to the olecranon of the right arm for the bicep muscles (Fig. 1c), and on the middle of the floor of the mouth for the cross-sectional area of the suprahyoid muscle (Fig. 1d). Since many previous studies used the cross-sectional area of the geniohyoid muscle to examine the relationship between sarcopenia and swallowing function, we measured cross-sectional area rather than thickness in this study^20^. The vertical diameters of the vastus intermedius and rectus femoris muscle complex was measured on the inner edge of their respective muscles to evaluate the thickness of the quadriceps, and the vertical diameter of the biceps muscle was measured on the inner edge of the muscle (Fig. 2). We adjusted the angle of the probe to obtain optimal perpendicular imaging of the underlying bone for correct measurements of the vastus intermedius and rectus femoris muscle complex and biceps muscles. The geniohyoid-mylohyoid muscle complex was measured on the floor of the mouth in the central sagittal plane as the cross-sectional area of the suprahyoid muscle. We adjusted the position of the probe to view the hyoid bone, mandible, and geniohyoid muscle on a single screen.

### Imaging of computed tomography

We measured the cross-sectional area of the psoas muscle using Aquilion PRIME (Canon Medical System Corporation, Tokyo, Japan) at 120 kV exposure. Manual tracing using CT imaging performed at the L3 level within a month before allo-HSCT was used to measure the cross-sectional areas of the bilateral psoas muscles.

### Statistical analysis

Inter-rater reliability is the degree of agreement between two or more evaluators, which considers the coherence of the implementation of a scoring system. Inter-rater reliability was calculated using the interclass correlation (ICC), which is a method to determine agreement and is expressed as a relative measure of the explained variance of the total random variance^21^. To estimate the validity of the ultrasound measurements, the level of correlation between pre-allo-HSCT ultrasound measurements and pre-allo-HSCT psoas muscle mass assessed using CT, which is known as the gold standard for evaluating muscle mass, was evaluated using Pearson correlation and a scatter plot^22^. Each median value of the ultrasound examination data assessed by three evaluators was used for analysis. Repeated measures analysis of variance (ANOVA) followed by a paired t-test with Bonferroni adjustment was used to analyze the differences in muscle mass at different times. Post hoc univariate linear regression analysis was performed to identify variables that contributed to the rate of change from baseline in each muscle mass at day 90 after allo-HSCT.

### Ethical declarations

The present study was approved by the ethics committee of Osaka International Cancer Institute (approval number 1706089013-2), and general consent was obtained from all patients at the time of operation.

## Results

Ninety-six patients were enrolled in this study. Among them, data from 68 recipients were available in the present study, of which 5 withdrew their consent and 17 dropped out due to relapse (n = 6), re-transportation (n = 2), and death (n = 9). The other six recipients were not able to undergo ultrasound evaluation at a certain point due to rejecting the evaluation due to physical and mental fatigue caused by GVHD or infection. The recipients’ characteristics are listed in Table 1. The mean age at allo-HSCT was 49 ± 12 years, and the diseases and donor sources were relatively well balanced. Acute GVHD was observed in 54 patients (79.4%). The mean length of hospital stay after allo-HSCT was 59.6 ± 23.9.

**Table 1:** Patients’ characteristics.

	n = 68
**Sex**	
Female	27 (39.7)
Male	41 (60.3)
Age, y	49.5 ± 12.4
Body mass index	21.8 ± 2.8
**Karnofsky performance status**	
100	48 (70.6)
90	19 (27.9)
80	1 (1.5)
**Disease n, (%)**	
AML	19 (27.9)
ALL/LBL	16 (23.5)
ML	17 (25.0)
MDS	8 (11.8)
CML/MPN	6 (8.8)
Other	1 (1.5)
**Stem cell source, n (%)**	
HLA-matched or 1 mismatched rPB	14 (20.6)
haploidentical rPB	15 (22.1)
uBM	18 (26.5)
uPB	2 (2.9)
CB	19 (27.9)
**Conditioning regimens**	
MAC	27 (39.7)
RIC	41 (60.3)
Period of neutrophil engraftment	15.68 ± 3.15
**Acute GVHD**	
Non	14 (20.6)
Grade I	27 (39.7)
Grade II	23 (33.8)
Grade III	3 (4.4)
Grade IV	1 (1.5)

AML, acute myeloid leukemia; ALL/LBL, acute lymphocytic leukemia/lymphoblastic lymphoma; ATLL, adult T-cell leukemia-lymphoma; ML, malignant lymphoma; MDS, myelodysplastic syndromes; CML/MPN, chronic myelogenous leukemia/myeloproliferative neoplasms, rPB: related peripheral blood, uBM: unrelated bone marrow, uPB: unrelated peripheral blood, CB: cord blood, MAC: myeloablative conditioning, RIC: reduced-intensity conditioning, GVHD: graft-versus-host disease.

### Inter-rater reliability

Table 2 shows the inter-rater reliability of the three evaluators for a total of 68 recipients, and thus, for a total of 273 evaluations. ICCs were 0.966, 0.977, 0.965, 0.964 in the biceps muscle; 0.962, 0.968, 0.975, 0.977 in the vastus intermedius muscle and rectus femoris muscle complex; and 0.899, 0.912, 0.897, 0.926 in the geniohyoid-mylohyoid muscle complex before and on days 30, 90 and 180 after allo-HSCT, respectively. All ICCs were high; thus, the measurements were reliable.

**Table 2:** Inter rater reliability of measurement of muscle mass using ultrasound.

	ICC (95% CI)
**Biceps muscle thickness**	
Before allo-HSCT	0.966 (0.953–0.976)
Days 30 after allo-HSCT	0.977 (0.968–0.984)
Days 90 after allo-HSCT	0.965 (0.950–0.976)
Days 180 after allo-HSCT	0.964 (0.947–0.975)
**Vastus intermedius muscle and rectus femoris muscle complex thickness**	
Before allo-HSCT	0.962 (0.948–0.973)
Days 30 after allo-HSCT	0.968 (0.955–0.977)
Days 90 after allo-HSCT	0.975 (0.964–0.983)
Days 180 after allo-HSCT	0.977 (0.966–0.984)
**CSA of geniohyoid-mylohyoid muscle complex**	
Before ffallo-HSCT	0.899 (0.863–0.927)
Days 30 after allo-HSCT	0.912 (0.879–0.937)
Days 90 after allo-HSCT	0.897 (0.856–0.929)
Days 180 after allo-HSCT	0.926 (0.895–0.950)

allo-HSCT: allogeneic hematopoietic stem cell transplantation. CSA: cross sectional area.

### Validity

**Figure 3:**
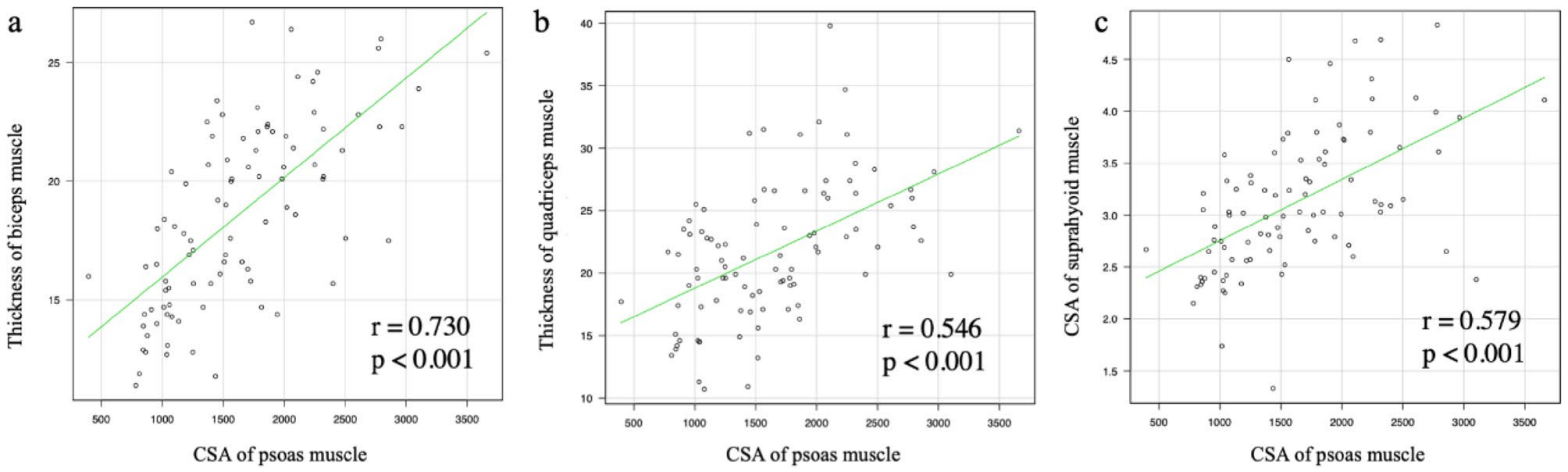
Scatter plots illustrating correlations between cross-sectional area of the psoas muscle and thickness of the biceps muscle (**a**), thickness of the quadriceps muscle (vastus intermedius muscle and rectus femoris muscle complex) (**b**), and cross-sectional area of the suprahyoid muscle (geniohyoid-mylohyoid muscle complex) (**c**).

Figure 3 shows the scatter plots illustrating the correlation between the cross-sectional area of the psoas muscle by CT and the median values of the ultrasound measurements as assessed by three evaluators, such as the thickness of the biceps muscle, vastus intermedius muscle and rectus femoris muscle complex, as well as the cross-sectional area of the geniohyoid-mylohyoid muscle complex before allo-HSCT in 96 recipients. The correlation scores were 0.730, 0.546, and 0.579 between the psoas muscle mass assessed with CT, the biceps muscle, the vastus intermedius muscle, the rectus femoris muscle complex, and the geniohyoid-mylohyoid muscle complex, respectively.

### Changes in muscle mass

**Figure 4:**
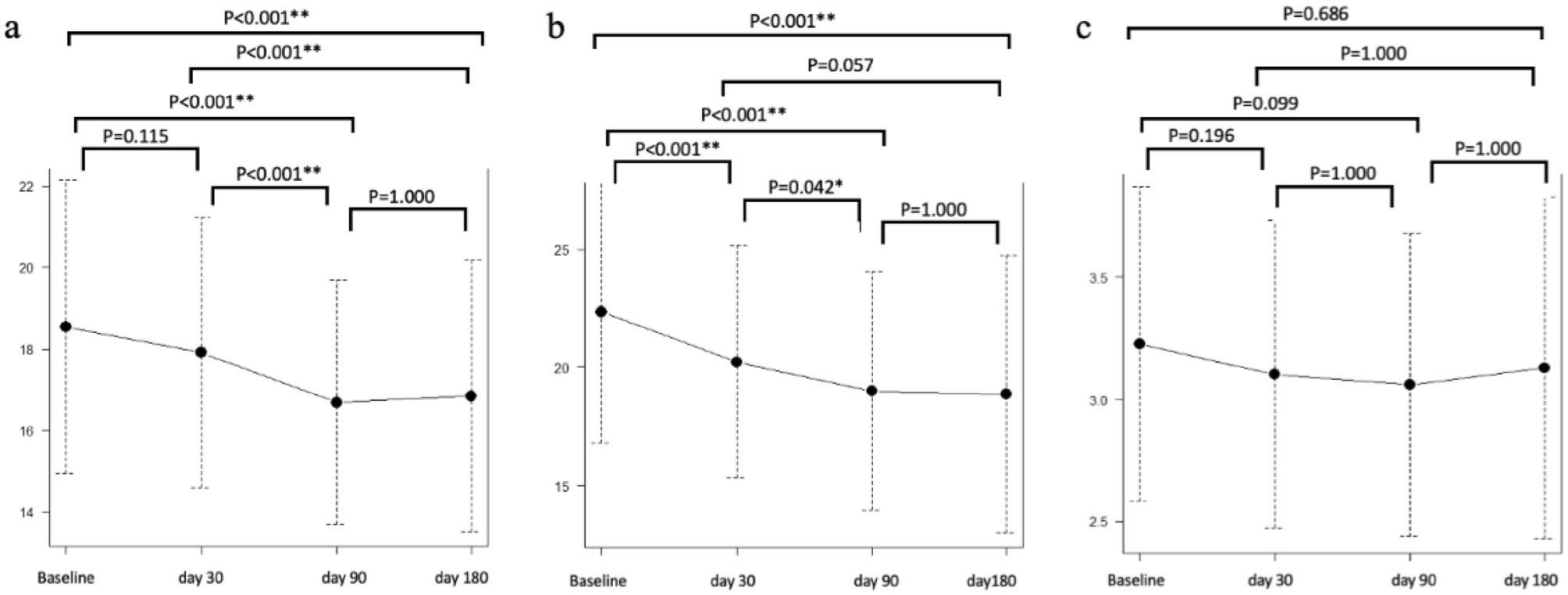
Changes of the thickness of the biceps muscle (**a**), thickness of the vastus intermedius muscle and rectus femoris muscle complex (**b**), and cross-sectional area of the geniohyoid-mylohyoid muscle complex (**c**). The mean ± SD muscle thickness and cross-sectional area at before and on days 30, 90 and 180 after allo-HSCT were as follows: 18.6 ± 3.6, 17.9 ± 3.3, 16.7 ± 2.9 and 16.9 ± 3.3 in the biceps muscle, 22.5 ± 5.6, 20.3 ± 4.9, 19.1 ± 5.1 and 18.9 ± 5.9) in the vastus intermedius muscle and rectus femoris muscle complex, and 3.22 ± 0.65, 3.10 ± 0.64, 3.05 ± 0.63 and 3.13 ± 0.70) in the geniohyoid-mylohyoid muscle complex, respectively. The thickness of the bicep muscles significantly declined at days 90 and days 180 after allo-HSCT from baseline and significantly declined at days 90 and days 180 after allo-HSCT from days 30. The thickness of the vastus intermedius muscle and rectus femoris muscle complex also significantly declined at days 30, 90 and 180 after allo-HSCT from baselines and significantly declined at days 90 from days 30. The level of statistical significance is marked with one asterisk (*) if P < 0.05 and two (**) if P < 0.01.

Figure 4 shows the change in the mean ± SD muscle thickness and cross-sectional area of the 68 recipients. The thickness of the bicep muscles significantly declined at days 90 and days 180 after allo-HSCT from baseline (18.6 ± 3.6 vs 16.7 ± 2.9, P < 0.001 and 16.9 ± 3.3, P < 0.001) and significantly declined at days 90 and days 180 after allo-HSCT from days 30 (17.9 ± 3.3 vs. 16.7 ± 2.9, P < 0.001 and 16.9 ± 3.3, P < 0.001). The thickness of the vastus intermedius muscle and rectus femoris muscle complex also significantly declined at days 30, 90 and 180 after allo-HSCT from baselines (22.5 ± 5.6 vs 20.3 ± 4.9, P < 0.001,19.1 ± 5.1, P < 0.001, and 18.9 ± 5.9, P < 0.001), and significantly declined at days 90 from days 30 (20.3 ± 4.9 vs 19.1 ± 5.1, P = 0.042). However, the difference was not statistically significant; the thickness of the biceps muscle muscles slightly recovered at 180 days after allo-HSCT compared with that at days 90 after allo-HSCT. There were no significant changes in the cross-sectional area of the geniohyoid-mylohyoid muscle complex. Supplementary Table S2 shows the post hoc univariate linear regression analysis. Steroid use was significantly associated with the rate of change in the biceps, vastus intermedius, and rectus femoris muscle complex thicknesses.

## Discussion

Our results suggest that ultrasonography is a valid and reliable method for assessing muscle mass in allo-HSCT recipients. In addition, this study also showed that ultrasound was able to detect changes in muscle mass, including the quadriceps and biceps muscles, in allo-HSCT recipients.

In this study, the measurements of muscle mass by ultrasound were highly reliable, with ICCs between 89.7% and 97.7%. Although muscle atrophy and edema occasionally occurred in allo-HSCT recipients, we obtained reliable results that were nearly equal to those of other studies^11,12,23^.

The validity of the ultrasound measurements was determined according to their correlation with psoas muscle mass as measured by CT. To assess whole-body muscle and sarcopenia, measurement of psoas muscle mass using CT is widely used as a gold standard^24^. Our findings revealed that psoas muscle mass measured by CT was highly correlated with the thickness of the biceps, vastus intermedius, and rectus femoris muscle complex, as well as the cross-sectional area of the geniohyoid-mylohyoid muscle complex, all measured by ultrasound. These results suggest that ultrasonography is a valid modality for assessing muscle mass and sarcopenia. In a meta-analysis of retrospective cohort studies, sarcopenia was found to be associated with non-relapse mortality and overall survival in patients after hematopoietic stem cell transplantation^25^. Thus, it is important for allo-HSCT recipients to assess muscle mass. Of these three muscles, we found that the thickness of the biceps muscle as measured by ultrasound was most correlated with the mass of the psoas muscle as measured by CT. This finding suggested that the biceps muscle might be more suitable for assessing muscle thickness using ultrasound.

In this study, we were able to detect changes in the thickness of the biceps and quadriceps muscles after allo-HSCT. All muscle masses decreased until day 90 after allo-HSCT. Some studies have reported that muscle mass decreases after allo-HSCT and autologous stem cell transplantation^2,26,27^. These studies showed a decrease in fat-free mass and muscle mass assessed between 17 and 100 days after transplantation. In this study, the thickness of the vastus intermedius muscle and rectus femoris muscle complex declined more sharply than the biceps muscle on days 30 after allo-HSCT, while the thickness of the biceps muscle declined more sharply from days 30 to 90 than the vastus intermedius muscle and rectus femoris muscle complex after allo-HSCT. In general, it has been reported that disuse or steroid-induced muscle atrophy occurs more frequently in lower limb muscles than in upper limb muscles^12,28^. Our results were different from those of previous studies^29^. After allo-HSCT, patients received physical therapy to strengthen their lower limb muscles; consequently, the mass of the lower limb muscle might recover more rapidly than that of the upper limb muscle. There were no significant changes in the cross-sectional area of the geniohyoid-mylohyoid muscle complex after allo-HSCT. Based on the findings that changes in the cross-sectional area of the geniohyoid-mylohyoid muscle complex were smaller than the changes in other muscles, the suprahyoid muscles might be less affected by disease, malnutrition, or steroid myopathy compared to other muscles.

All muscle masses evaluated tended to recover or stop declining from day 90 to 180 after allo-HSCT. Physical activity has been reported to increase at 3 and 6 months after allo-HSCT, with exercise programs having an effect on muscle strength and aerobic capacity^6^. Moreover, most recipients who received allo-HSCT exhibited malnutrition at the time of discharge, but the number of recipients was decreased upon long-term follow-up^30,31^. Therefore, three months after allo-HSCT, the recipients’ activities and nutritional intake may be essential for improving muscle mass atrophy.

At the 180 days follow-up, we obtained data of 69% (68 patients) in this study. These results are higher than those of a previous study that evaluated 40.7% of recipients who met the inclusion criteria for physical assessment and muscle mass on BIA^2^. Evaluating physical function is sometimes difficult for allo-HSCT recipients due to medical problems or fatigue. In addition, assessing muscle mass with CT or BIA is also difficult due to the patient being in an isolation room or having edema^2^. Thus, obtaining a high rate of follow-up data in this study suggested that ultrasound measurement could be a good alternative method for evaluating muscle mass.

In conclusion, we found that ultrasound measurement of muscle mass is a reliable and valid tool for allo-HSCT recipients and may be useful particularly when we were not able to assess physical ability. In addition, the results of changes in muscle mass suggested that muscle mass tended to decrease after allo-HSCT but started to recover from day 90 after allo-HSCT. Further studies are needed to clarify the association between ultrasound measurements and physical ability.

## Supplementary Information

Supplementary Information.
