# Supplementary material for: Reliability and validity of ultrasound to measure of muscle mass following allogeneic hematopoietic stem cell transplantation
Source: Sci Rep. 2022 Jan 27;12:1538. doi: 10.1038/s41598-022-05577-1 (PMC8795441; doi:10.1038/s41598-022-05577-1)
Supplement: Supplementary file 1 — Supplementary Information. [file 41598_2022_5577_MOESM1_ESM.docx]

**Supplemental information**

Supplementary Table.

Supplementary Table S1. Inter rater reliability of measurement of muscle mass using ultrasound. A total of 92, 86, 79, and 70 patients were able to undergo ultrasound measurements at baseline and on days 30, 90, and 180, respectively.

|  |  |  | ICC (95% CI) |
| --- | --- | --- | --- |
| Biceps muscle thickness | | |  |
|  | Before allo-HSCT | | 0.968 (0.954 - 0.978) |
|  | Days 30 after allo-HSCT | | 0.978 (0.968 - 0.985) |
|  | Days 90 after allo-HSCT | | 0.961 (0.943 - 0.974) |
|  | Days 180 after allo-HSCT | | 0.964 (0.947 - 0.975) |
| Vastus intermedius muscle and rectus femoris muscle complex thickness | | |  |
|  | Before allo-HSCT | | 0.962 (0.945 - 0.974) |
|  | Days 30 after allo-HSCT | | 0.971 (0.958 - 0.980) |
|  | Days 90 after allo-HSCT | | 0.976 (0.965 - 0.984) |
|  | Days 180 after allo-HSCT | | 0.977 (0.966 - 0.984) |
| CSA of geniohyoid-mylohyoid muscle complex | | |  |
|  | Before allo-HSCT | | 0.895 (0.852 - 0.928) |
|  | Days 30 after allo-HSCT | | 0.905 (0.866 - 0.935) |
|  | Days 90 after allo-HSCT | | 0.883 (0.832 - 0.922) |
|  | Days 180 after allo-HSCT | | 0.926 (0.895 - 0.950) |

allo-HSCT: allogeneic hematopoietic stem cell transplantation

Supplementary Table S2. Post hoc univariate linear regression analysis was performed to identify variables that contributed to rate of change in each muscle mass at day 90 after allo-HSCT from baseline.

|  | β coefficient | 95% CI | P value |
| --- | --- | --- | --- |
| Biceps muscle | | |  |
| Age | -0.11 | -0.30 to 0.08 | 0.272 |
| Female | 5.71 | 0.94 to 10.47 | 0.019 |
| BMI | -0.69 | -1.54 to 0.15 | 0.106 |
| MAC | -0.29 | -5.26 to 4.67 | 0.906 |
| Presence of acute GVHD | 2.11 | -3.87 to 8.11 | 0.483 |
| Use of steroid | -5.77 | -10.71 to -0.83 | 0.023 |
|  |  |  |  |
| Vastus intermedius muscle and rectus femoris muscle complex | | |  |
| Age | -0.31 | -0.64 to 0.01 | 0.063 |
| Female | 0.44 | -8.13 to 9.01 | 0.918 |
| BMI | -0.23 | -1.72 to 1.26 | 0.760 |
| MAC | 2.63 | 0.20 to 5.06 | 0.034 |
| Presence of acute GVHD | 6.06 | -4.20 to 16.32 | 0.242 |
| Use of steroid | -14.43 | -22.56 to -6.30 | <0.001 |
|  |  |  |  |
| CSA of geniohyoid-mylohyoid muscle complex | | |  |
| Age | -0.22 | -0.57 to 0.13 | 0.216 |
| Female | 8.32 | -0.38 to 17.0 | 0.061 |
| BMI | 0.48 | -1.06 to 2.03 | 0.531 |
| MAC | 8.78 | 0.10 to 17.46 | 0.047 |
| Presence of acute GVHD | 4.60 | -6.16 to 15.36 | 0.396 |
| Use of steroid | 3.11 | -6.10 to 12.33 | 0.502 |

BMI: body mass index, MAC: myeloablative conditioning, GVHD: graft-versus-host disease, CSA: cross sectional area

Supplementary Figure S1


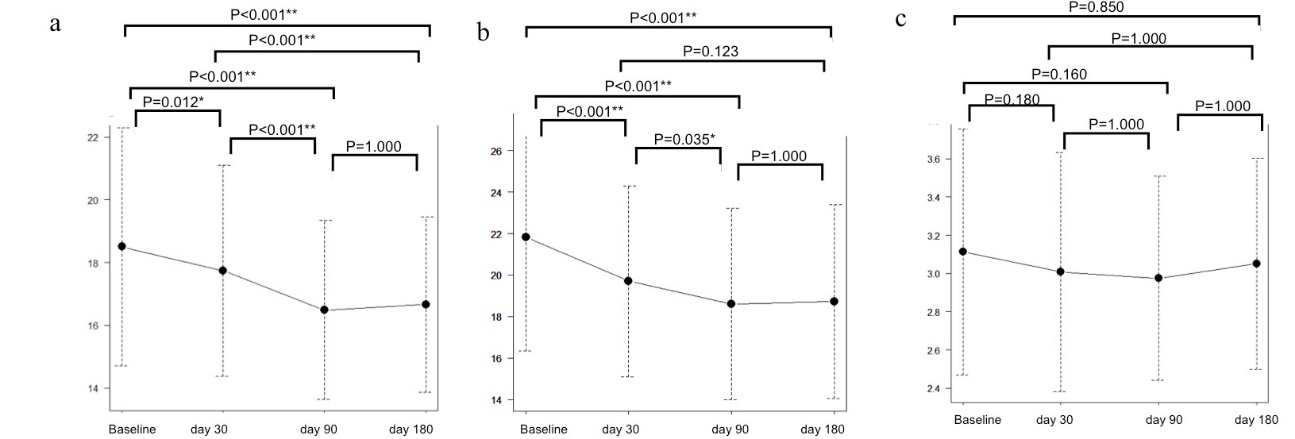


**Fig. S1** Changes of the thickness of the biceps muscle (a), thickness of the vastus intermedius muscle and rectus femoris muscle complex (b), and cross-sectional area of the geniohyoid-mylohyoid muscle complex (c). A total of 92, 86, 79 and 70 patients were able to undergo ultrasound measurements at baseline and on days 30, 90, and 180, respectively. The mean ± SD muscle thickness and cross-sectional area at before and on days 30, 90 and 180 after allo-HSCT were as follows: 18.5 ± 3.8, 17.7 ± 3.6, 16.5 ± 3.1 and 16.7 ± 3.3 in the biceps muscle, 21.8 ± 5.5, 19.7 ± 4.9, 18.6 ± 5.1 and 18.7 ± 5.5) in the vastus intermedius muscle and rectus femoris muscle complex, and 3.11 ± 0.65, 3.01 ± 0.67, 2.98 ± 0.59 and 3.05 ± 0.67) in the geniohyoid-mylohyoid muscle complex, respectively. The thickness of the bicep muscles significantly declined at days 90 and days 180 after allo-HSCT from baseline and significantly declined at days 90 and days 180 after allo-HSCT from days 30. The thickness of the vastus intermedius muscle and rectus femoris muscle complex also significantly declined at days 30, 90 and 180 after allo-HSCT from baselines and significantly declined at days 90 from days 30. The level of statistical significance is marked with one asterisk (*) if P<0.05 and two (**) if P<0.01
